# Testing Behavior Change Techniques to Increase Physical Activity in Middle-Aged and Older Adults: Protocol for a Randomized Personalized Trial Series

**DOI:** 10.2196/43418

**Published:** 2023-06-14

**Authors:** Ciaran P Friel, Patrick L Robles, Mark Butler, Challace Pahlevan-Ibrekic, Joan Duer-Hefele, Frank Vicari, Thevaa Chandereng, Ken Cheung, Jerry Suls, Karina W Davidson

**Affiliations:** 1 Institute of Health System Science Feinstein Institutes for Medical Research Northwell Health New York, NY United States; 2 Mailman School of Public Health Columbia University New York, NY United States

**Keywords:** behavior change techniques, N-of-1, personalized trials, physical activity, behavior change, aging, quality of life, feasibility, acceptability, effectiveness, web-based, intervention, text, email, survey

## Abstract

**Background:**

Being physically active is critical to successful aging, but most middle-aged and older adults do not move enough. Research has shown that even small increases in activity can have a significant impact on risk reduction and improve quality of life. Some behavior change techniques (BCTs) can increase activity, but prior studies on their effectiveness have primarily tested them in between-subjects trials and in aggregate. These design approaches, while robust, fail to identify those BCTs most influential for a given individual. In contrast, a personalized, or N-of-1, trial design can assess a person’s response to each specific intervention.

**Objective:**

This study is designed to test the feasibility, acceptability, and preliminary effectiveness of a remotely delivered personalized behavioral intervention to increase low-intensity physical activity (ie, walking) in adults aged 45 to 75 years.

**Methods:**

The intervention will be administered over 10 weeks, starting with a 2-week baseline period followed by 4 BCTs (goal-setting, self-monitoring, feedback, and action planning) delivered one at a time, each for 2 weeks. In total, 60 participants will be randomized post baseline to 1 of 24 intervention sequences. Physical activity will be continuously measured by a wearable activity tracker, and intervention components and outcome measures will be delivered and collected by email, SMS text messages, and surveys. The effect of the overall intervention on step counts relative to baseline will be examined using generalized linear mixed models with an autoregressive model that accounts for possible autocorrelation and linear trends for daily steps across time. Participant satisfaction with the study components and attitudes and opinions toward personalized trials will be measured at the intervention's conclusion.

**Results:**

Pooled change in daily step count will be reported between baseline and individual BCTs and baseline versus overall intervention. Self-efficacy scores will be compared between baseline and individual BCTs and between baseline and the overall intervention. Mean and SD will be reported for survey measures (participant satisfaction with study components and attitudes and opinions toward personalized trials).

**Conclusions:**

Assessing the feasibility and acceptability of delivering a personalized, remote physical activity intervention for middle-aged and older adults will inform what steps will be needed to scale up to a fully powered and within-subjects experimental design remotely. Examining the effect of each BCT in isolation will allow for their unique impact to be assessed and support design of future behavioral interventions. In using a personalized trial design, the heterogeneity of individual responses for each BCT can be quantified and inform later National Institutes of Health stages of intervention development trials.

**Trial Registration:**

clinicaltrials.gov NCT04967313; https://clinicaltrials.gov/ct2/show/NCT04967313

**International Registered Report Identifier (IRRID):**

RR1-10.2196/43418

## Introduction

The health benefits of physical activity (PA) are wide ranging, from reducing all-cause mortality and cardiovascular disease risk [1,2] to improving mental health [3]. However, most Americans fail to meet recommended levels of PA, with a prevalence of inactivity increasing as people age [4,5]. This age-related decline in activity is concerning as the benefits of PA are particularly potent as we get older [6]. The development of effective PA interventions such that everyone meets national guidelines remains a central goal, but recent research findings have shown that even small increases in PA offer health benefits [1,2]. Furthermore, a nonlinear dose response exists between PA volume and disease risk reduction, with the greatest drop in risk seen in very inactive older adults who increase PA by even a small amount [2]. Low-intensity PA, such as walking, has been identified as an effective and well-accepted modality, with increases of as little as 1000 steps per day found to lower the risk of mortality [2].

While the evidence demonstrating health benefits for older adults from even small amounts of PA is reassuring [2], questions remain on the most effective ways to encourage any positive change in PA among this population. While there have been numerous PA interventions for older adults, few have shown more than modest improvements [7]. One plausible reason is that many of these studies have been conventional randomized controlled trials (RCTs) with little consideration for active components of behavior interventions, no consideration for heterogeneity of treatment effects, and a mean outcome effect calculated, for example, pre-post change in daily step count for the hypothetical “average” older adult. While between-subjects RCTs are the gold standard of research trials, this design cannot calculate individual heterogeneity of treatment effects, as individuals are only exposed to 1 condition—the single condition to which they were randomized [8]. Even in clinically significant between-subject RCTs, some participants increase in activity, while others show no change or even decrease in activity in response to the intervention.

To address these concerns, some RCT designs focus on within-person behavior change with exposure to multiple interventions. Personalized trial designs, otherwise known as “N-of-1” trial designs, use a within-subject experimental approach to evaluate the outcomes of different interventions specific to that subject [9,10]. For example, a personalized trial can provide a quantitative estimate of the effects of distinct behavioral interventions on a single participant by observing the participant continuously over a given period with the different interventions occurring at scheduled time points. Observing the effects of multiple interventions can determine the quantitative effect of each specific intervention on an individual participant. This means effect sizes for each intervention can be confirmed for that participant rather than relying on aggregate findings that include data from other participants.

Personalized trials have been used to study fatigue, depression, back pain, and preventive behaviors [11,12], but they have not been widely employed in real-world clinical practice because the delivery of such an approach has been perceived as overly burdensome for clinicians and patients [13]. However, with the advent of smartphones, cloud computing, and biometric or activity trackers, platforms can now be developed to conduct N-of-trials more feasibly. N-of-1 trials using this technology can allow researchers to deliver behavioral interventions and obtain real-world data remotely, often in real time.

Extensive research has attempted to untangle those key elements, known as behavior change techniques (BCTs), that contribute to successful changes in behaviors such as smoking, diet, or PA [14,15]. From this body of research, we first focused on those that have been associated with improvements in PA [16] and then narrowed our intervention selection down to 4 BCTs that were most strongly associated with a change in walking and could be delivered effectively using our trial design. The 4 BCTs selected are goal setting, self-monitoring of behavior, action-planning, and feedback on behavior.

This study protocol is designed to evaluate the feasibility and acceptability of a randomized personalized trial that will serially deliver 4 BCTs in random order to promote low-intensity PA. The trial will recruit 60 adults aged 45-75 years. The protocol will integrate wearable technology (ie, Fitbit) capable of measuring participant activity (eg, step count and heart rate) with SMS text messaging and survey delivery via smartphone such that the research team can conduct the study remotely and collect data continuously. The study’s primary outcomes will be within-person changes in daily steps and participant satisfaction with personalized trial approach components. Secondary outcomes include self-efficacy for walking and participant attitudes and opinions toward personalized trials.

## Methods

### Design

The purpose of this study is to determine the feasibility, acceptability and preliminary effectiveness of using personalized or N-of-1 methods to increase low-intensity walking by 2000 steps per day for 5 days each week in a remote research study. Participants can accumulate these additional 2000 steps across the course of the day and do not have to do so in a single bout. The study will recruit Northwell employees aged 45 to 75 years old and use 4 BCTs that have been shown as effective in encouraging increases in PA.

**Figure 1:**
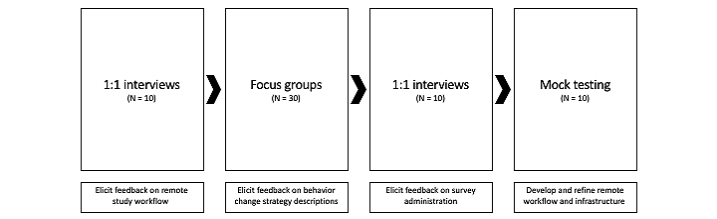
Pretrial user testing.

The study design comprises 2 main parts. Part 1 entails a series of 4 phases (see Figure 1) where participants will be recruited to help develop the content and processes for the second main part, which is the intervention—a personalized trial series. Up to 60 participants will participate in the initial series of 4 phases undergoing focus groups, interviews, and surveys to elicit feedback concerning the clarity of instructions and convenience of messaging. They will provide feedback on key aspects of study design, including BCT choices, BCT descriptions, materials, and electronic delivery methods such as SMS text messaging and email receipt. Up to 10 of the 60 participants will engage with study materials and procedures as “mock participants,” interacting remotely and providing feedback about the ease and acceptability of study procedures to further refine study materials and procedures.

**Figure 2:**
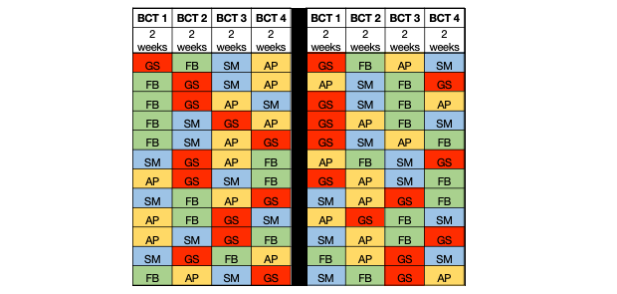
Intervention sequences. AP: action planning; BCT: behavior change technique; FB: feedback on behavior; GS: goal setting; SM: self-monitoring.

The second main part of the study entails an intervention, where 60 participants (who were not involved in the earlier phases of focus groups and user testing) will be recruited for a 10-week personalized trial assessing the impact of 4 BCTs, assigned in random order, on their walking behavior. Participants will be sent a Fitbit Charge 4 tracker that will monitor the number of steps taken per day. At the end of each 2-week intervention period, participants will complete a survey stating their satisfaction with the specific single BCT delivered during that time. Each BCT will be presented for 2 weeks during the 8-week active intervention in a randomly selected order (see Figure 2). Our study statistician, who is not involved in recruitment, will generate a list of assignments using block randomization, which they will then provide to our project manager. Participants will be assigned a BCT sequence if they complete the baseline and meet all adherence criteria.

At the end of their 10-week trial, participants will receive a detailed summary of their observed data and a final, comprehensive satisfaction survey regarding the overall personalized trial design. The summary will help participants learn more about their responses to each of the 4 BCTs. The satisfaction survey will inform investigators about the feasibility of personalized study design for future research and clinical applications.

### Participants, Recruitment, and Screening

Potential participants will be Northwell Health employees who are community residing, 45 to 75 years old, healthy, report that they walk regularly without health and safety issues, and express an interest in participating in a personalized trial to increase their walking.

The study will use Northwell Health’s existing employee networks to recruit eligible participants. Potential participants may be recruited via the following measures: (1) advertisement emails to those who previously expressed an interest in participating in a personalized trial, (2) flyers circulated within the Northwell Health network, and (3) Northwell Health employee social media sites such as Northwell’s employee-only Facebook group.

Interested participants will first be invited to complete a “Screening Survey” where they must respond to inclusion and exclusion criteria. Clinical research coordinators will review these data and determine whether a potential participant is eligible to participate.

### Ethics Approval

The protocol and consent processes have been approved by the institutional review board (IRB; 20-1166) at Northwell Health’s Feinstein Institutes for Medical Research. The study protocol conforms to the ethical guidelines of the 1975 Declaration of Helsinki as reflected in a priori approval by the institution’s human research committee, Northwell Health Institutional Review Board. The consent document will contain all the elements of informed consent required by applicable federal regulation for the protection of human subjects and elements of authorization required by the Health Insurance Portability and Accountability Act Privacy Rule.

### Consent Process

The consent will be presented electronically as an IRB-approved information sheet for the pretrial phases and as a full, IRB-approved informed consent form requiring a digital signature for the intervention. As consent for the intervention will be obtained remotely, the electronic platform will be designed such that the consent form is easy to navigate. We will use interactive, electronic-based technology to present information about the research in a variety of different modalities to facilitate understanding before participants document their consent (eg, print and video formats). The consent document will contain all the elements of informed consent required by applicable federal regulation for the protection of human subjects and elements of authorization required by the Health Insurance Portability and Accountability Act Privacy Rule and will begin with a concise and focused presentation of the key information that is most likely to assist participants in understanding the reasons why they may or may not want to participate in the research. These descriptions will be carefully scripted in language that persons with a sixth-grade education can understand; key information will include a brief description of the participant’s proposed involvement, potential risks, and benefits of participation. Voluntary participation affirming participants that they have the right to stop at any time without impact to their employment or care will be emphasized. Potential participants must demonstrate comprehension of the informed consent form by responding to 4 questions about the trial prior to documenting their consent. Their electronic informed consent will be obtained prior to enrollment in the project for those individuals who are eligible. Participants will receive a copy of their signed informed consent form via SMS text message. Since consents are obtained electronically, they will remain in our Research Electronic Data Capture database (Vanderbilt University) indefinitely. The IRB-approved informed consent is included in Multimedia Appendix 1.

### Privacy and Confidentiality

Given that study activities involve no more than minimal risks encountered in daily life (ie, increased time spent walking by healthy, working individuals), this study does not require oversight by a Data Safety and Monitoring Committee. Rather, the study has received National Institutes of Health approval for a safety monitor. This safety monitor will be assigned to periodically review and evaluate project data for participant safety, scientific integrity, and trial progress. There is no additional risk with using a Fitbit activity monitor for research as compared to using the device as a consumer, including mild skin irritation (ie, contact dermatitis) which occurs among a small proportion of users. One risk of taking part in this study is the possibility of a loss of confidentiality or privacy. The risk of loss of confidentiality will be minimized by securely storing data including protected health information in a Northwell-approved database and minimizing the use of protected health information.

### Participant Compensation

Participant compensation will vary according to the part of the study in which the participant is involved. Participants in the 4 pretrial phases will receive 5000 points credited to their Northwell MyRecognition account (roughly equivalent to US $25) for attending a focus group, participating in an interview, or completing their mock study procedures.

For the intervention, involving 10 weeks of study participation, participants will receive a pay card valued at US $100 for completing all study requirements. Additionally, participants in the intervention will be able to keep their Fitbit, valued at US $150.

### Focus Group Procedures

Up to 60 participants will participate in the first 4 pretrial phases of the study. They will be invited to participate in 1-on-1 interviews (up to 20 participants), focus groups (up to 30), or mock testing (up to 10). Participants will provide feedback on key aspects of study design such as BCT descriptions, study workflow remotely (ie, remote screening, informed consent process, and demographic questionnaire), and remote survey administration (ie, satisfaction and self-efficacy surveys).

Participants in the focus groups and 1-on-1 interviews will be presented with study materials and potential aspects of study designs. Participants will then be asked questions on the clarity and readability of that content—for example, IRB-approved questions such as “Do the descriptions make sense?” and “What would motivate someone to participate in this study?” These focus groups and interviews will be conducted by a trained facilitator with a principal investigator and note-taker present. Ten of the 60 participants will engage in mock testing of study materials and procedures, providing feedback to the study team on the acceptability of these procedures. This will allow the study team to develop and refine the workflow and infrastructure remotely.

### Personalized Trial Series Procedures

As noted earlier, an intervention in the form of a personalized trial series will follow the pretrial phases This series will comprise a single trial for each eligible participant that includes a 2-week baseline period and 8 weeks of study intervention, testing 1 BCT at a time each for a 2-week period. During baseline, participants must maintain 80% compliance with Fitbit wear (ie, device is worn a minimum of 10 waking hours on a given day) and responses to questionnaires to be eligible for the intervention period.

### Intervention Onboarding

If participants consent, they will receive an “Onboarding Survey” to complete. This form will collect the participant’s demographic information and necessary contact information so that the study team can ship the activity tracker and study materials. Once research staff confirm that the participant has completed the required onboarding tasks, an initial study kit, which includes a Fitbit Charge 4 and printed materials to guide the participant through the study, will be shipped to them. The printed materials will instruct participants how to download an app to their personal smartphone so they can use the Fitbit as well as instructions on how to charge and synchronize it. Anonymous study accounts will be created to protect the privacy of the participant.

### Intervention Baseline

Participants will be mailed a study kit including a Fitbit Charge 4 device and printed study materials. The study materials will include the participant’s start date and instructions on how to download the Fitbit app to their smartphone so they can pair the Fitbit device. All baseline periods will begin on a Monday, and no more than 20 participants will be permitted to begin on the same day.

The baseline period will be 2 weeks in duration. Potential participants will not receive any text message BCT interventions during baseline, but they will be asked to wear their Fitbit all day and night (as tolerated), sync their Fitbit device at least every 2 days, and respond to 1 text message per day to ensure they are receiving communications.

At the end of the baseline period, a clinical research coordinator will review individual adherence to Fitbit wear and responsiveness to the daily surveys. Adherence to Fitbit wear will be defined as having at least 600 minutes (10 hours) of heart rate activity per day. SMS text message adherence will be defined as having responded to the daily message. Baseline participants who do not achieve at least 80% adherence to both Fitbit wear and SMS text message responses during the 2-week baseline period will be withdrawn from the study. Those who maintain at least 80% adherence will be randomized to 1 of 24 sequences of 4 BCTs received 1 at a time, switching every 2 weeks (see Figure 2). Enrollment will continue until up to 60 participants have been randomized.

### Main Intervention—BCT Sequences

Participants who successfully complete the baseline period will receive a random sequence of BCT interventions over the next 8 weeks during which a BCT message is delivered daily for a 2-week period. For example, goal-setting messages will be sent for 2 weeks, then self-monitoring prompts for 2 weeks, and so on for action planning and feedback. On the day before each new 2-week period, participants will be sent a message describing which BCT is coming next. BCT messages will be automatically delivered each day, and the participant’s own data from baseline and the previous day will be used to generate messages. A description of BCTs employed and sample messages is provided in Table 1.

Participants will also receive a biweekly survey to assess their satisfaction with the BCT intervention sent over those 2 weeks and a self-efficacy survey assessing their confidence in their ability to walk for varying periods of time. Participants may receive additional SMS text messages to those outlined above with reminders to sync their data if needed.

**Table 1:** Description of behavior change technique intervention content.

Content	Modalities	Sample SMS text message prompt
Goal setting	SMS text message is delivered daily to encourage the participant to set a goal of beating their average daily step count during baseline.	“Is your goal today to walk an extra 2000 steps more than your baseline average?”
Self-monitoring of behavior	Ongoing self-monitoring is prompted by a daily text message delivered each morning. The baseline average is included in the message.	“Check your Fitbit dashboard for yesterday’s activity. Did you take an extra 2,000 steps more than your baseline average?”
Feedback on behavior	An SMS text message is delivered each morning stating whether or not the goal had been met on the day prior (Fitbit step data are collected remotely)	“You met your goal yesterday. You walked 2000 steps more than your baseline average.” “You did not meet your goal yesterday of walking at least an extra 2,000 steps more than your baseline average”
Action planning	An SMS text message is delivered each morning encouraging the participant to plan for an additional walking session. The baseline average is included in the message.	“Take one minute and plan how, where, and when today you can walk an extra 2000 steps over your baseline.”

### Postintervention

Upon completion, participants in the intervention will receive an individual report that presents changes in their own walking behavior in relation to their selected BCTs. Participants will also receive a final survey concerning their satisfaction with the personalized trial design and their attitudes and opinions toward personalized trials. After receiving the report, clinical research coordinators will notify the participant that they have completed the study, remind them of the US $100 pay card for completing the study, and instruct them to unlink the Fitbit device from the anonymous study account. Participants will also be invited to contact the study team for any remaining questions.

### Retention Plans During Intervention

The study has a well-formulated action plan for participant retention: when a participant fails to sync their device or respond to questionnaires for 2 consecutive days, they will receive an automated message notifying them that they have dipped below the benchmark. If improvement is not seen within another 2 days, the clinical research coordinator will reach out to the participant by text/phone/email and troubleshoot issues. Retention rates will be reported in weekly team meetings and, if needed, the study will institute additional incentives such as a weekly regret lottery to ensure compliance with study procedures and retention of study participants. A regret lottery is a method adopted from behavioral economics where participants are entered into a money- or prize-based lottery for which prize receipt is contingent upon participants’ achievement of protocol adherence goals [17].

### Measures and Analysis: Data Integrity

The study data manager will be responsible for ensuring the integrity of data collected and the preparation of the data for statistical analysis. The data manager is available to provide data to the safety office upon the safety monitor’s request in between monthly reports.

Responses from interviews and focus groups in the pretrial phases will be tabulated into documented themes for consideration in the intervention design, such as acceptable levels of participant burden (eg, optimal number of SMS text messages per week) and consensus on design features including the content of messaging.

For missing data, the plan is to invest in collecting data as completely as possible. Given the preliminary work to refine the BCT description and study design prior to the 60 personalized trials, we anticipate minimal missing data in recording participant responses. Intention-to-treat analysis will be used. In the unlikely case of missing data, we will examine the missingness pattern and its association with any phenotypes, thus identifying potential confounders and use multiple imputation approaches.

### Primary Outcomes

#### Within-Person Change in Daily Steps

The Fitbit device continuously collects the number of steps taken by each participant. These data can be examined at a daily level, and the study will aggregate daily step counts for both the baseline and intervention periods [18]. This will allow the research teams to compare the daily step count between each period to see if any change occurs.

#### Participant Satisfaction With Personalized Trial Components

At the end of the intervention period and after receiving their individual study report, the participant will receive a satisfaction survey comprising nine items with 4-point Likert scales. Each item is preceded by the prompt “How satisfied were you with the following?” and the 4-point scale ranges from “Not at all satisfied” to “Very satisfied.”

### Secondary Outcomes

#### Self-Efficacy for Walking

Self-efficacy has been shown to predict longer-term behavior, mediating the relationship between positive experience and PA [19]. At the end of the baseline period and every 2 weeks during the intervention period, participants will complete the Self-Efficacy for Walking Scale [20]. It is a validated 10-item survey assessing the participant’s confidence in their ability to walk for a given duration. The phrase “I BELIEVE THAT I CAN WALK” precedes all of the 10 sentences with each one specifying a different amount of time (eg, “For 5 minutes at a moderately fast pace without stopping”). The first item in the survey specifies 5 minutes of walking and the amount increases by 5 minutes with each subsequent item such that the final item specifies 50 minutes of walking. The questions are scored on an 11-point scale, which ranges from “Not at all confident” to “Highly confident” with “Moderately confident” in the middle.

#### Participant Attitudes and Opinions About the Personalized Trial Implementation

To measure attitudes and opinions about personalized trial implementation, participants will be asked to respond to an 11-item survey on a 7-point scale. This survey is modified from the widely used system usability scale [21]. Each item asks about some aspect of the personalized trial. For example, 1 item reads “The materials I received in the mail were clear and easy to follow” and another reads “I needed to learn a lot of things before I could get going with my personalized trial.” These statements will prompt the participant to indicate how much that statement applies to them; on one end of the scale reads, “Strongly disagree,” and the other reads, “Strongly agree.”

### Primary Analyses

Means and SDs for daily steps will be reported for the baseline period and for each BCT intervention period. The effect of the overall intervention relative to baseline will be examined using generalized linear mixed models with an AR(1) (autoregressive order of 1) model that accounts for possible autocorrelation and linear trends for daily steps across time. The overall intervention effect will be tested based on whether the coefficient of the time effect is positive. Additional generalized linear mixed models will be examined to identify the effects of individual BCTs on daily steps. For these AR(1) models, fixed effects will be specified for each BCT intervention and random effects will be specified for each participant. We will adopt a novel Bayesian distributed lagged model to further explore the extent of carryover effects [22]. For participant satisfaction with personalized trial components, responses to the questions will be reported with means and SDs. The distribution of participant responses for each item assessing satisfaction will be displayed using a bar graph.

### Secondary Analyses

Means and SDs for self-efficacy will be reported for the baseline period and for each BCT intervention period. To identify whether self-efficacy for walking mediates the effect of the BCT intervention on daily steps, we will perform the analysis in 3 steps. First, we will estimate the effects of the BCT intervention on self-efficacy. This step will be examined using linear mixed-effects models, which include a random participant effect, fixed effects for the BCT intervention, a fixed time effect, and fixed BCT-time interaction. Next, we will estimate the effect of the BCT intervention on daily steps using linear regression. Finally, we will estimate the indirect effect of the BCT intervention on daily steps via the proposed mediating variable of self-efficacy. This will use natural effects models for effect decomposition into direct effect and indirect effect, mediated by an increase in self-efficacy, relative to baseline [23,24]. Participant attitudes and opinions toward personalized trials will be reported with means and SDs. Bar graphs will be used to display the distribution of participant responses for each item.

### Sample Size Justification

Sample size was based on the primary outcome for within-person change in daily steps due to the overall BCT intervention. As this is a feasibility trial, sample size estimates were calculated based on the effect of the overall intervention, not the individual BCT interventions. The effect size is obtained from a previous study [25]. Using a paired *t* test with the effect size of 0.404 and type I error of 0.05 (2-sided), we obtain a power of 80% when comparing the number of steps during BCT period with the baseline period for 50 subjects. We expect a change in step of 2000 between the baseline and BCT periods. We obtained a pooled standard error of 4989.77 from Pinto et al [25], from a SE of 2476 (preintervention period) and 3555 (postintervention period). To achieve a total of 50 participants with complete data, under an assumed 15% attrition, we anticipate randomizing up to 60 participants. This sample size will ensure adequate size to obtain a preliminary assessment of this trial's feasibility, which will be determined by the effectiveness of the overall BCT intervention on PA behavior and participant scores on the satisfaction survey. The number of assessment measures and treatment repetitions per trial are based on expert recommendations by the study statistician and their estimations about the maximal duration of the trial to maintain patient engagement.

## Results

The project was funded in 2021, and enrollment was completed in February 2022. Data analysis is currently underway, and the first results are expected to be submitted for publication in June 2023.

## Discussion

### Principal Findings

This study will evaluate the feasibility and acceptability of a randomized personalized trial to promote low-intensity PA in middle-aged and older adults. Sixty adults aged 45-75 years old will receive 4 BCTs, one at a time, for a 2-week each. The study will be conducted remotely, using automated SMS text messaging and survey delivery, and wearable technology to monitor participant activity. The study’s primary outcomes will be within-person changes in daily steps and participant satisfaction with personalized trial approach components. This study’s findings will deliver insights into 2 main areas of research interest. First will be the feasibility of delivering a series of individual BCTs in a personalized design to influence PA behavior. Most studies to date have incorporated multiple BCTs in their intervention, which limits our understanding of each BCT’s specific impact. Second is the feasibility and acceptability of the personalized trial design as measured by participant satisfaction and completion of trial measures. This approach offers the promise of unique findings that will be used to support later National Institutes of Health stages of intervention development trials. Personalized trials are just one of several innovative trial design approaches being used to address the limitations of classic RCTs. The multiphase optimization strategy and the sequential multiple assignment randomized trial are 2 prominent examples of the many adaptive approaches now being used to facilitate more effective trials and garner greater insights about the component parts that make an intervention successful [26-29]. What is unique about the personalized trial approach is its ability to deliver insights regarding the heterogeneity of effect from a given intervention and support personalized recommendations for each individual [30].

### Limitations

There are a number of potential limitations to this study. The first is that there may have been some reactivity to wearing the Fitbit device that could have impacted the baseline step counts. In a future study, where the goal of the study is to examine reactivity to devices and calibrate the effectiveness of commercially available activity trackers like Fitbit, research-grade accelerometers (which do not display steps or other metrics) could be used along with Fitbit devices for a longer duration of time to enhance the data collection. The second limitation is that there is no maximum threshold of activity rather participants will be included if they felt they could benefit from walking more each day. This may lead to the inclusion of participants who are already quite active and could result in a ceiling effect and limit generalizability to less active adults. The third limitation is that the participants may turn on features in the Fitbit app that could deliver additional BCTs (such as adding friends or joining challenges) not part of the intervention. A final limitation is that all participants will be employees at the same health care organization, thus limiting the generalizability of findings.

### Conclusions

This remotely delivered study will contribute broadly to the science of PA behavior change in middle-aged and older adults. BCTs are seen as key components of behavior change, but the influence of specific BCTs is not well understood [31,32]. The findings from this study will provide insights into the extent to which individual BCTs are effective in changing PA behavior. Furthermore, it will demonstrate if the influence of BCT on walking is consistent across participants or if large individual heterogeneous treatment effects exist [32]. How older adults engage with the technical components of this trial, such as wearable devices and remote onboarding, will inform current practice and offer a foundation for the development of larger personalized trials. In conclusion, this study offers a vision of conducting future series of trials where personalized data derived from wearable sensors, rather than generic guidelines, are used to inform the selection of intervention components for each individual.

## Appendix

Multimedia Appendix 1 Example of change in steps across behavior change technique periods.

## Abbreviations

BCT: behavior change technique
IRB: institutional review board
PA: physical activity
RCT: randomized controlled trial

## Data Availability

This trial was preregistered on ClinicalTrials.gov (NCT04967313) and data will be made available on the Open Science Framework [33].
