# Supplementary figures and images for: Testing Behavior Change Techniques to Increase Physical Activity in Middle-Aged and Older Adults: Protocol for a Randomized Personalized Trial Series
Source: JMIR Res Protoc. 2023 Jun 14;12:e43418. doi: 10.2196/43418 (PMC10337349; doi:10.2196/43418)

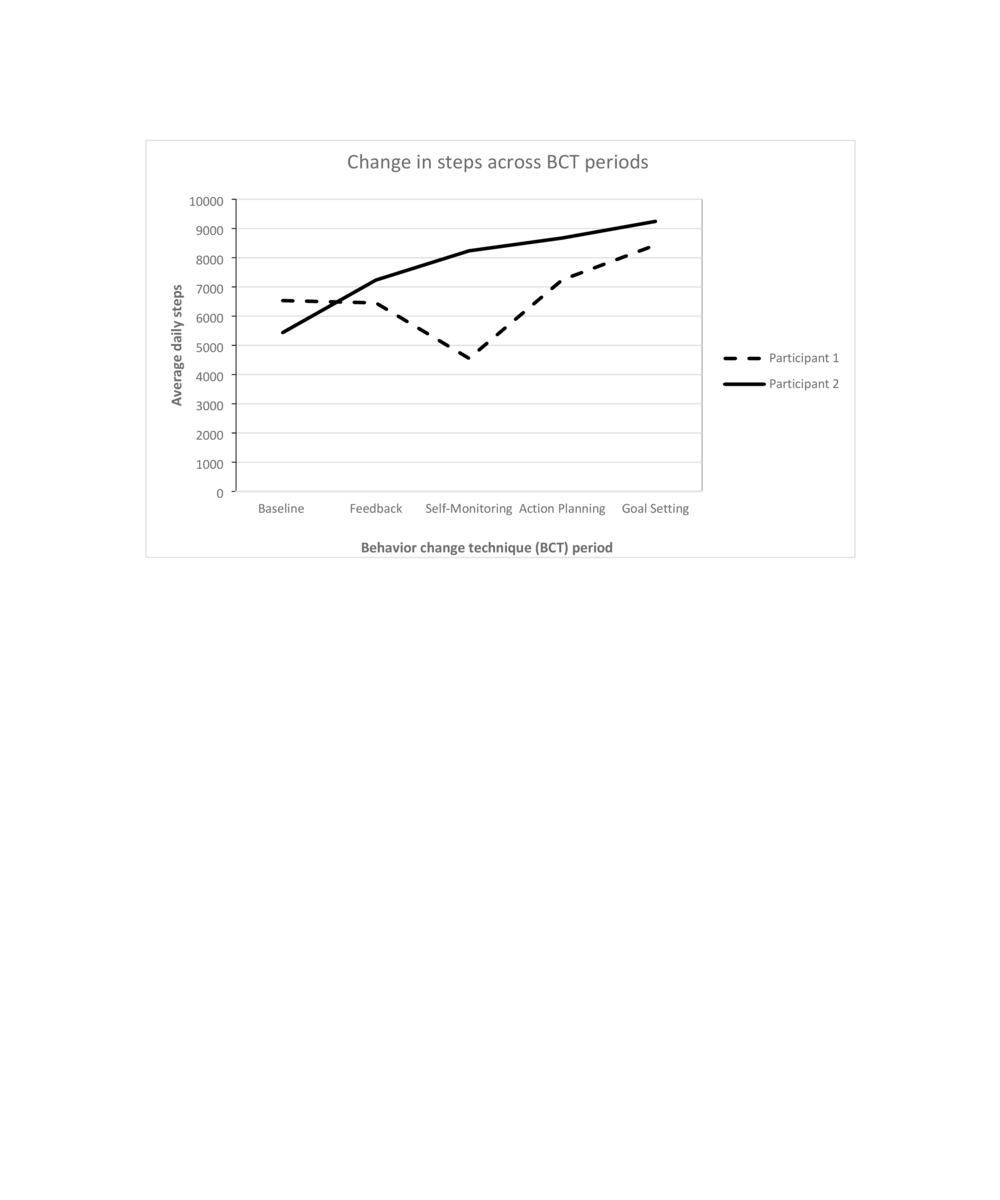

Supplement: Multimedia Appendix 1 [file resprot_v12i1e43418_app1.png]
